# Genome analysis and exploration of *Bacillus subtilis* XH-10, a potential biocontrol strain

**DOI:** 10.3389/fmicb.2026.1896430

**Published:** 2026-07-30

**Authors:** Youpin Xu, Qihang Wan, Yuehuan Hu, Yuke Huang, Ling Zhong, Jin Jiang, Zhixiong Chen

**Affiliations:** 1 School of Biological and Environmental Engineering, Guiyang University, Guiyang, Guizhou, China; 2 Key Laboratory of Southwest China Wildlife Resources Conservation (Ministry of Education), College of Life Science, China West Normal University, Nanchong, Sichuan, China Nanchong Academy of Agricultural Sciences, Nanchong, Sichuan, China; 3 School of Electrical Engineering and Electronic Information, Xihua University, Chengdu, Sichuan, China; 4 Guizhou Key Laboratory of Agricultural Biosecurity, Guiyang University, Guiyang, Guizhou, China; 5 Nanchong Academy of Agricultural Sciences, Nanchong, Sichuan, China

**Keywords:** *Bacillus subtilis*, biocontrol, Fusarium head blight of wheat, secondary metabolites, whole-genome sequencing

## Abstract

**Background:**

Wheat is an important staple crop that has received widespread attention in recent years. *Fusarium* head blight (FHB) seriously affects wheat yield and quality, and also causes mycotoxin contamination.

**Objective:**

To explore green control strategies, this study focused on the mining of biocontrol resources against FHB.

**Results:**

A bacterial strain, XH-10, showing strong antagonistic activity against *Fusarium graminearum* (the major causal agent of FHB), was isolated using the dilution plating method and dual culture assay. Based on morphological characteristics and 16S rDNA gene sequence analysis, strain XH-10 was identiffed as *Bacillus subtilis*. Antimicrobial tests using the fermentation broth showed that the growth of the pathogen was inhibited in the presence of the XH-10 fermentation broth, accompanied by hyphal malformation, swelling, breakage, and a signiffcant reduction in spore production. Pathogenicity assays revealed that the addition of the XH-10 fermentation broth reduced the virulence of the *F. graminearum* PH-1 toward wheat. Whole-genome analysis showed that the genome size of strain XH-10 is 4.1 Mb, with a GC content of 43.78%, encoding 4,233 genes. Seven secondary metabolite biosynthetic gene clusters (BGCs) were predicted, including those responsible for the synthesis of known antimicrobial substances such as surfactin, bacillaene, fengycin, bacillibactin, subtilosin A, and bacilysin. Functional annotation further revealed the presence of siderophore biosynthesis gene clusters, glycoside hydrolase genes including chitinase, and sporulation-related genes.

**Conclusion:**

The XH-10 exhibits promising biocontrol potential against wheat FHB, and its biocontrol mechanisms may involve the production of multiple antimicrobial compounds and the secretion of cell wall-degrading enzymes. This study provides a valuable microbial resource and theoretical basis for the development of biocontrol agents against wheat FHB.

## 1 Introduction

Wheat (*Triticum aestivum* L.) is a cereal crop of the genus *Triticum*, rich in starch, protein, dietary fiber, and various vitamins and minerals. It serves as a staple food source for approximately 35% of the global population. Currently, the annual global wheat production has reached 800 million tons, cultivated on approximately 219 million hectares of land (Audenaert et al., 2010), with major production areas in Asia, Europe, North and South America, Australasia, and other regions (Xu et al., 2022). It is estimated that wheat production will need to increase by about 60% over the next three decades to meet the demands of a growing world population (Petronaitis et al., 2021).

However, wheat production is increasingly threatened by diseases. Among them, FHB, caused by *Fusarium* spp., is one of the most destructive diseases affecting wheat. The disease primarily damages wheat spikes, leading to spike blight, shriveled kernels, and contamination with mycotoxins such as deoxynivalenol (DON). Under severe conditions, FHB can cause yield losses ranging from 30 to 50% (Horo et al., 2026; Xu et al., 2022). Recent studies have indicated a clear increasing trend in the prevalence of FHB. The pathogen spreads rapidly via wind and rain during the heading and flowering stages, resulting in substantial yield reductions (Xu et al., 2022). Moreover, *Fusarium* spp. can survive for long periods in crop residues and soil, making the disease particularly difficult to manage (Mourelos et al., 2024). Therefore, research aimed at the prevention and control of FHB is of great significance for ensuring safe wheat production.

Currently, due to the lack of wheat varieties that combine both high yield and effective resistance to FHB, chemical control remains the primary strategy for managing this disease. However, the long-term and repeated use of chemical fungicides can easily lead to the emergence of resistant pathogen strains, cause environmental pollution, degrade soil structure, and result in chemical residues in wheat grains. Since wheat is a major staple food crop, these issues conflict with the requirements for green and safe production of food crops (Naqvi et al., 2025; Audenaert et al., 2010).

Biological control, which employs beneficial microorganisms and their metabolites to suppress plant diseases, offers advantages such as environmental compatibility and a lower risk of resistance development, making it an important approach for achieving green and sustainable agriculture (Kashyap et al., 2025). Currently, the biocontrol bacteria most commonly used against plant diseases include species of *Bacillus* (Liao et al., 2026), *Pseudomonas* (Xu et al., 2025), and *Paenibacillus* (Wen et al., 2026). These bacteria have become a focus of biocontrol research due to their ability to produce stress-resistant spores, as well as a wide range of antimicrobial substances and enzymes. Although biological control has been applied to many plant diseases, further research is still needed specifically on the control of wheat FHB.

*Bacillus* spp., as an important group of biocontrol microorganisms, exhibit antimicrobial activity closely linked to their biosynthetic potential for secondary metabolites. In recent years, with the rapid development of high-throughput sequencing technologies and the continuous improvement of bioinformatics tools, whole-genome sequencing has become a core approach for elucidating the biocontrol mechanisms of *Bacillus* (Ortiz et al., 2026). Studies have shown that biosynthetic gene clusters (BGCs) encoding secondary metabolites account for approximately 5–10% of the *Bacillus* genome. These gene clusters are not only conserved at the genus level but also exhibit significant strain-specificity (Naser et al., 2025).

Ortiz et al. systematically summarized the secondary metabolic characteristics of the *B. subtilis* group and pointed out that lipopeptides (e.g., surfactin, fengycin, iturin), polyketides (e.g., bacillaene, difficidin), and siderophores (e.g., bacillibactin) represent the most core antimicrobial compounds in this group (Ortiz et al., 2026). Building on this, multiple studies have further revealed the BGC composition and antifungal functions of specific strains. For instance (Zhang et al., 2026) performed whole-genome sequencing of *B. velezensis* JZ and identified multiple antimicrobial lipopeptide BGCs, including those for surfactin, fengycin, bacillaene, bacillibactin, and macrolactin H, and further validated the antimicrobial activity of six secondary metabolites, including carvone, using metabolomics (Zhang et al., 2026). An integrative analysis of *B. velezensis* GFB08 indicated that fengycin and bacillomycin D are the major antifungal compounds, exhibiting an additive effect against the pathogen (Cho et al., 2025). Liu et al. predicted 14 BGCs in the genome of *B. velezensis* YNK-FB0059 and validated its broad-spectrum antagonistic activity against *F. graminearum*, with inhibition rates ranging from 77.8 to 88.6% (Liu et al., 2026). Amjad et al. and Nikolaeva et al. identified multiple PKS/NRPS-type BGCs in *B. subtilis* strains BAGL and NGII, respectively, covering key antimicrobial substances such as surfactin, fengycin, bacillibactin, and subtilosin A (Amjad et al., 2026; Nikolaeva et al., 2025).

Collectively, these studies demonstrate that the combined application of whole-genome sequencing and genome mining enables efficient identification of gene clusters encoding antimicrobial substances in biocontrol bacteria. The systematic mining of these genetic resources provides a robust molecular basis for the rational design and development of novel biological pesticides and has become a research hotspot in the field of biological control.

In this study, a biocontrol strain XH-10 with high antagonistic activity against *F. graminearum* was isolated. Based on morphological and molecular biological analyses, strain XH-10 was identified as *B. subtilis*. The fermentation broth of strain XH-10 inhibited mycelial growth, sporulation, and pathogenicity of the FHB pathogen, and also exhibited broad-spectrum antifungal activity. Modern sequencing technologies were comprehensively applied to analyze the whole genome of strain XH-10, predict its biosynthetic gene clusters for antimicrobial secondary metabolites, and mine functional genes related to biocontrol. This study aims to elucidate the genomic features and potential biocontrol mechanisms of strain XH-10, thereby providing a theoretical foundation for the development of microbial agents against wheat FHB.

## 2 Materials and methods

### 2.1 Experimental materials and culture conditions

The following culture medium components, including glucose (A501991), agar (A505255), carboxymethyl cellulose sodium (A501427), KH₂PO₄ (A600456), MgSO₄·7H₂O (A500864), yeast extract (A610961-0500), and peptone (A505247), were all purchased from Sangon Biotech Co., Ltd. (Shanghai, China). In addition, potato infusion powder (FA0270, Solarbio, Beijing, China) and NH₄NO₃ (2,015,042,301, Chengdu Kelong Chemicals Co., Ltd., Sichuan, China) were also used for medium preparation.

The tested strain of *Fusarium graminearum* (PH-1) was provided by the State Key Laboratory of Crop Stress Biology in Arid Areas, Northwest A&F University. It was cultured on PDA plates (potato infusion 7 g/L, glucose 20 g/L, agar 20 g/L, autoclaved at 121 °C for 15 min) at 28 °C for 3 days for subsequent experiments.

Preparation of PH-1 conidia: Mycelial plugs (5 mm in diameter) of PH-1 grown on PDA were transferred to CMC sporulation medium (carboxymethyl cellulose sodium 15 g, NH₄NO₃ 1 g, KH₂PO₄ 1 g, MgSO₄·7H₂O 0.5 g, yeast extract 1 g, distilled water to 1 L, autoclaved at 121 °C for 50 min) to induce sporulation for 3–5 days.

Conidial germination: The collected conidia of PH-1 were added to YEPD liquid medium (yeast extract 3 g, peptone 10 g, glucose 20 g, distilled water to 1 L) to adjust the concentration to 1 × 10^5^ spore/mL. The culture was incubated in a constant temperature shaker (Shanghai Zhichu Instrument Co., Ltd., ZQZY-CS9, Shanghai, China) at 28 °C. Samples were taken at 0, 2, 4, 8, 10, 12, and 24 h for microscopic (Nexcope, NE910, Ningbo, China) observation.

Wheat material: The susceptible wheat variety Nanmai 660, provided by the Nanchong Academy of Agricultural Sciences (Nanchong, China), was planted at the scientific research experimental base of China West Normal University.

Biocontrol strain XH-10: Strain XH-10 was isolated by our laboratory. The strain was streaked onto PDA plates and cultured at 28 °C for 2 days, then stored at 4 °C for subsequent use.

Preparation of XH-10 fermentation broth: Under sterile conditions, a single colony of strain XH-10 on PDA medium was picked and inoculated into 2 mL of PDB medium, then incubated at 28 °C in a shaker at 180 rpm for 2 days to prepare the seed culture. A 200 μL aliquot of seed culture (OD₆₀₀ = 0.092) was inoculated into 50 mL PDB medium and incubated at 28 °C with shaking at 180 rpm for 2 days. The fermentation broth reached an OD₆₀₀ of 0.314, and was then centrifuged at 12,000 rpm for 10 min. The supernatant was collected as the fermentation broth.

Main reagents: Bacterial Genomic DNA Extraction Kit (Macklin, B917890, Shanghai, China), Gram Staining Kit (Macklin, 77,730-1KT-F, Shanghai, China), 2 × Rapid Taq Master Mix (Vazyme, P222-01, Nanjing, China). Primer synthesis was completed by Youkang Biotechnology Co., Ltd. (Hangzhou, China). 16S rDNA sequencing was performed by Sangon Biotech Co., Ltd. (Shanghai, China). The whole-genome sequencing of XH-10 was completed by Bena Sequencing Co., Ltd. (Wuhan, China).

### 2.2 Isolation, identification, and antagonistic screening of strain XH-10

Soil samples were collected from the rhizosphere of healthy wheat plants in diseased fields at China West Normal University (Nanchong, China). Microorganisms were isolated from the soil samples using the dilution plating method. Single colonies were picked and repeatedly streaked for purification to obtain pure cultures.

The dual culture method was used for antagonistic screening. A mycelial plug (0.04 cm^2^) of PH-1 was inoculated at the center of a PDA plate. Four 1 μL aliquots of the XH-10 bacterial suspension were dropped at the four vertices of a square around the center. The plates were incubated at 25 °C for 5 days, after which the inhibition rate was calculated. Control plates were inoculated with PH-1 alone and with sterile water dropped at the four vertices. The inhibition rate was calculated as follows: Inhibition rate (%) = [(Average radius of control group−Radius of experimental group) / Average radius of control group] × 100%.

The morphological structure of strain XH-10 was observed by Gram staining. The 16S rDNA gene sequence of strain XH-10 was amplified using universal bacterial primers 16S-R/F. The PCR product was sent to Sangon Biotech Co., Ltd. for sequencing. The obtained sequences were subjected to BLAST (BLAST: Basic Local Alignment Search Tool) analysis, and a phylogenetic tree was constructed using MEGA 5.0^1^ for species identification.

### 2.3 Analysis of the antagonistic activity of XH-10 against PH-1

Effect on mycelial growth: To analyze the effect of XH-10 fermentation broth on the mycelial growth of PH-1, the fermentation broth was added to PDA medium to prepare plates with final concentrations of 50, 5, and 0.5% (v/v). A mycelial plug (approximately 0.04 cm^2^) of PH-1 was inoculated at the center of each plate. After incubation at 28 °C for 5 days, the colony diameter was measured, and the inhibition rate was calculated using the formula: Inhibition rate (%) = [(Average diameter of control group−Average diameter of experimental group) / Average diameter of control group] × 100%. Control plates were prepared without the addition of XH-10 fermentation broth. The mycelial morphology and structure on the above plates were also observed microscopically.

Effect on sporulation: To analyze the effect of XH-10 on sporulation of PH-1, the fermentation broth was added to CMC medium at final concentrations of 50, 5, and 0.5% (v/v). Three mycelial plugs (5 mm in diameter) of PH-1 were inoculated into each flask. Control groups were prepared without the addition of XH-10 fermentation broth. After incubation at 28 °C in a constant temperature shaker for 5 days, the spore production was quantified.

Effect on conidial germination: To analyze the effect of XH-10 on conidial germination of PH-1, YEPD liquid medium was used to induce conidial germination. The XH-10 fermentation broth was added to YEPD liquid medium at final concentrations of 50, 5, and 0.5% (v/v). A PH-1 spore suspension was then added to achieve a final concentration of 1 × 10^5^ spore/mL. Microscopic observation and quantification were performed at 2, 4, 8, 12, and 24 h.

All experiments were performed in triplicate with three biological replicates.

### 2.4 Effect of XH-10 on the pathogenicity of PH-1

Leaf infection assay: To analyze the effect of XH-10 on the infectivity of PH-1, wheat leaf infection assays were performed using the wound inoculation method. Healthy wheat leaves at the 5-week growth stage were wounded every 2 cm using a pipette tip. The XH-10 fermentation broth was added to the PH-1 spore suspension to achieve final concentrations of 50, 5, and 0.5% (v/v), with a final PH-1 spore concentration of 1 × 10^5^ spore/mL. The suspension was used to inoculate the wounded leaves. Disease development was observed after 5 days. Control plants were inoculated with the spore suspension without XH-10 fermentation broth. Fifteen wounds were inoculated per treatment.

Spike inoculation assay: Further experiments were conducted using the single-floret inoculation method on wheat spikes. The test spore suspensions were prepared as described above. A 10 μL aliquot of each suspension was inoculated into the middle spikelet of wheat spikes at the flowering stage. After maintaining high humidity for 4 days, the plants were cultured for an additional 10–16 days, after which disease development was observed. Six wheat spikes were inoculated per treatment.

All experiments were performed in triplicate with three biological replicates.

### 2.5 Whole-genome sequencing and assembly of XH-10

A total of 1.0 g of XH-10 cells at the logarithmic growth phase (collected by centrifugation) was used for genomic DNA extraction. The cells were ground with liquid nitrogen, followed by the addition of SDS (Mabioway, MAR-3639, Xian, China) lysis buffer [containing appropriate amounts of proteinase K (Biolab, SY0001, Beijing, China) and *β*-mercaptoethanol (Thermo Fisher Scientific, 21,985,023, Waltham, MA, USA)]. The mixture was gently inverted to ensure complete lysis. After lysis, the sample was centrifuged at 12,000 rpm for 5–10 min at 4 °C, and the supernatant was collected. The supernatant was extracted twice with a chloroform-isoamyl alcohol mixture (24:1, v/v) (Sigma-Aldrich, 25,668, St. Louis, MO, USA). DNA was precipitated by adding isopropanol, washed twice with 75% ethanol, and further purified using OMEGA purification columns (OMEGA BIO-TEK, D6492-01, Norcross, GA, USA) and Ampure XP magnetic beads (Beckman Coulter, A63881, Brea, CA, USA).

DNA quality was assessed using a Nanodrop spectrophotometer (Thermo Fisher Scientific, ND-2000/ND-1000, Waltham, MA, USA), a Qubit fluorometer (Thermo Fisher Scientific, Qubit 2.0/3.0/4.0, Waltham, MA, USA), and agarose gel electrophoresis. After passing quality control, the DNA sample was sent to Bena Technology Co., Ltd. (Wuhan, China) for whole-genome sequencing. A 1D sequencing library was constructed using the SQK-LSK110 kit (Oxford Nanopore Technologies, SQK-LSK110, Oxford, UK), and a small fragment library was constructed using the VAHTS® Universal Plus DNA Library Prep Kit (Vazyme, ND617, Nanjing, China). Sequencing was performed on the Nanopore PromethION platform (Oxford Nanopore Technologies, Oxford, UK) and the Illumina NovaSeq 6,000 platform (Illumina, USA).

Subsequently, Unicycler software (version 0.5.0) (Wick et al., 2016) was used for hybrid assembly of the two-generation sequencing data, with the k-mer parameter set to 31. The assembly strategy first utilized the high-accuracy Illumina sequencing data (Q30 quality value proportion >85%) for initial assembly, based on which a high-quality bacterial genome scaffold was constructed.

The whole-genome sequence data reported in this study have been deposited in the Genome Warehouse of the National Genomics Data Center (Beijing Institute of Genomics, Chinese Academy of Sciences / China National Center for Bioinformation) under accession number GWHJMXG01000000 and are publicly accessible at https://ngdc.cncb.ac.cn/gwh.

### 2.6 Gene prediction and functional annotation of the XH-10 genome

The prediction and annotation of functional elements in the genome were performed as follows. Prokka software (Version 1.14.6) (Seemann, 2014) was used for coding gene prediction of the assembled genome. This software integrates a series of functional element prediction tools, including Prodigal, Aragorn, RNAmmer, and Infernal for coding gene, tRNA, rRNA, and miscRNA prediction, respectively (Seemann, 2014).

For other functional elements, CRISPR sequences were predicted using MinCED (Version 0.4.2)^2^. Genomic islands were predicted using IslandPath (Version 1.0.6) (Hsiao et al., 2003). Prophage sequences were predicted using the online tool PhiSpy (Version 4.2.21) (Akhter et al., 2012). Although the repeat sequence content in prokaryotic genomes is relatively low, RepeatMasker (Version 4.1.2-p1) (Nishimura, 2000) was used for repeat sequence detection. The antiSMASH program (Version 6.0.0) (Blin et al., 2021) was used to predict secondary metabolite biosynthetic gene clusters, and clusters with integrity <70% were excluded as false positives. Insertion sequences were predicted using BLAST software combined with the ISfinder database^3^. Promoter sequence analysis was performed using PromPredict (v1)^4^.

Functional annotation of the XH-10 genome adopted a multi-database annotation strategy, covering KEGG (including KEGG Pathway) (v2024-05-27)^5^, Gene Ontology (GO) (v2024-05-29)^6^, Pfam (v37)^7^, COG (v1.0)^8^, TIGRFAMs (v15.0)^9^, and NR databases (v2024-02-07)^10^. The predicted gene sequences were compared against these functional databases using BLAST+ software (v2.11.0+)^11^ to obtain preliminary functional annotation results. Additionally, HMMER software (v3.3.2) (Finn et al., 2011) was used for protein domain-level functional annotation based on the Pfam and TIGRFAMs databases.

### 2.7 Statistical analysis

Statistical analyses were performed using one-way analysis of variance (ANOVA) in SPSS software (v17.0)^12^. Mean comparisons were conducted using Duncan’s multiple range test (Duncan, 1955), with statistical significance set at *p* ≤ 0.05.

## 3 Results

### 3.1 Screening and identification of antagonistic bacterium XH-10 against PH-1

**Figure 1:**
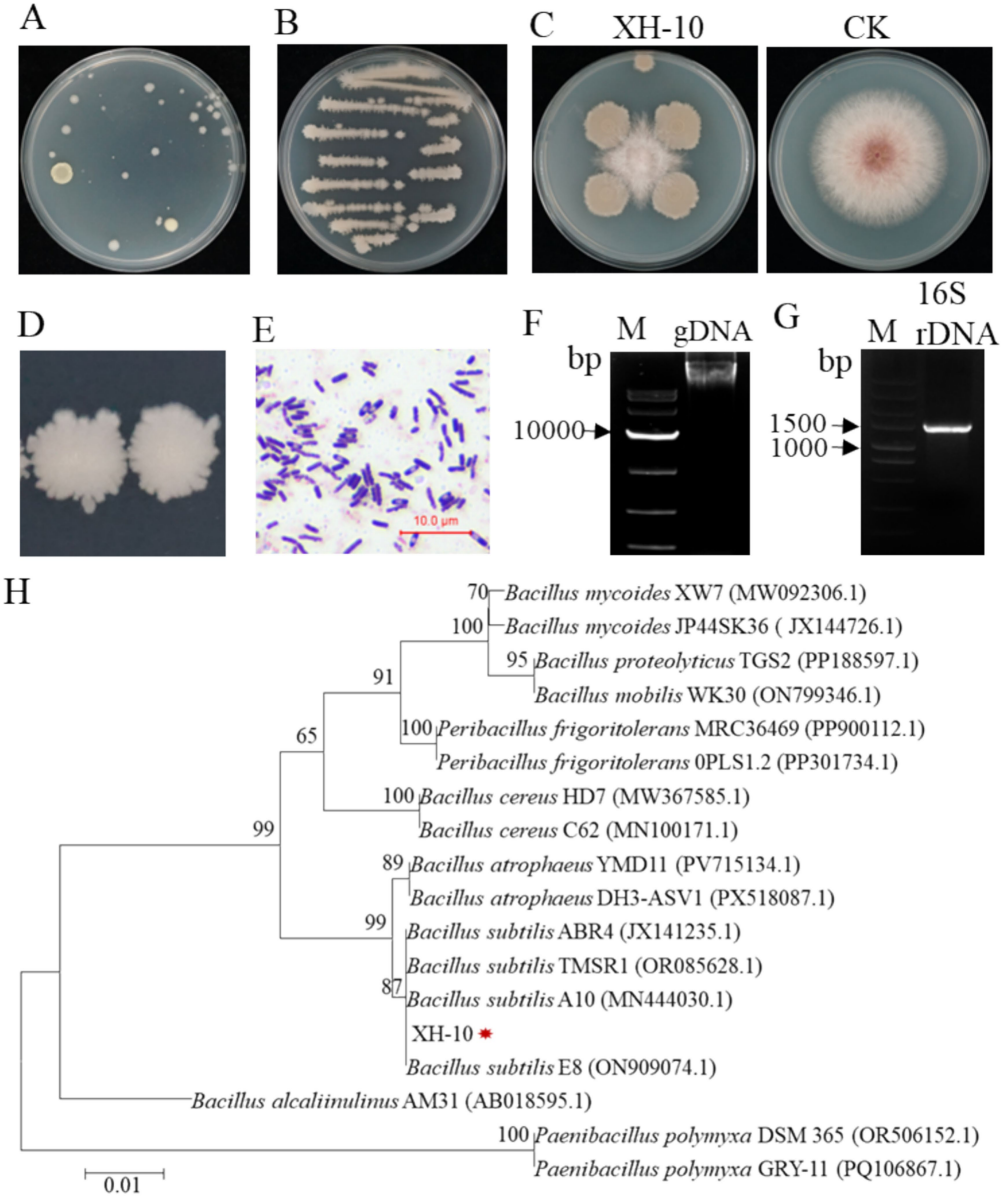
Screening and identification of the biocontrol bacterium XH-10 against wheat Fusarium head blight (FHB). **(A)** Isolation of XH-10 from soil samples; **(B)** Purification of XH-10 by streak plating; **(C)** Dual culture assay between XH-10 and PH-1 (CK: sterile water instead of XH-10); **(D)** Colonial morphology of XH-10 on PDA medium; **(E)** Gram staining result of XH-10; **(F)** Genomic DNA electrophoresis of XH-10 (M: DNA marker); **(G)** PCR amplification electrophoresis of the 16S rDNA gene of XH-10 (M: DNA marker); **(H)** Phylogenetic tree constructed based on the 16S rDNA sequence of XH-10.

To explore biocontrol resources with potential for managing *Fusarium* head blight (FHB), a soil sample designated XH-10 was collected from the experimental base of China West Normal University. Biocontrol microorganisms were isolated and purified using the dilution plating method combined with continuous streaking (Figures 1A,B). The purified culture XH-10 was subjected to dual culture assay with PH-1. The results showed that XH-10 exhibited antagonistic activity against PH-1 compared to the control (Figure 1C), with a mycelial inhibition rate of 63.63%.

To determine its taxonomic status, morphological identification of XH-10 was performed. It was observed that colonies of XH-10 on PDA plates exhibited irregular protrusions at the edges, with a dry and wrinkled surface and a slight yellowish color (Figure 1D). Further Gram staining revealed that the strain was Gram-positive (Figure 1E). Genomic DNA of XH-10 was extracted (Figure 1F), and identification was carried out by 16S rDNA sequence analysis (Figure 1G). Strain XH-10 was identified as *B. subtilis* (Figure 1H).

### 3.2 Antagonistic activity of XH-10 fermentation broth against PH-1

**Figure 2:**
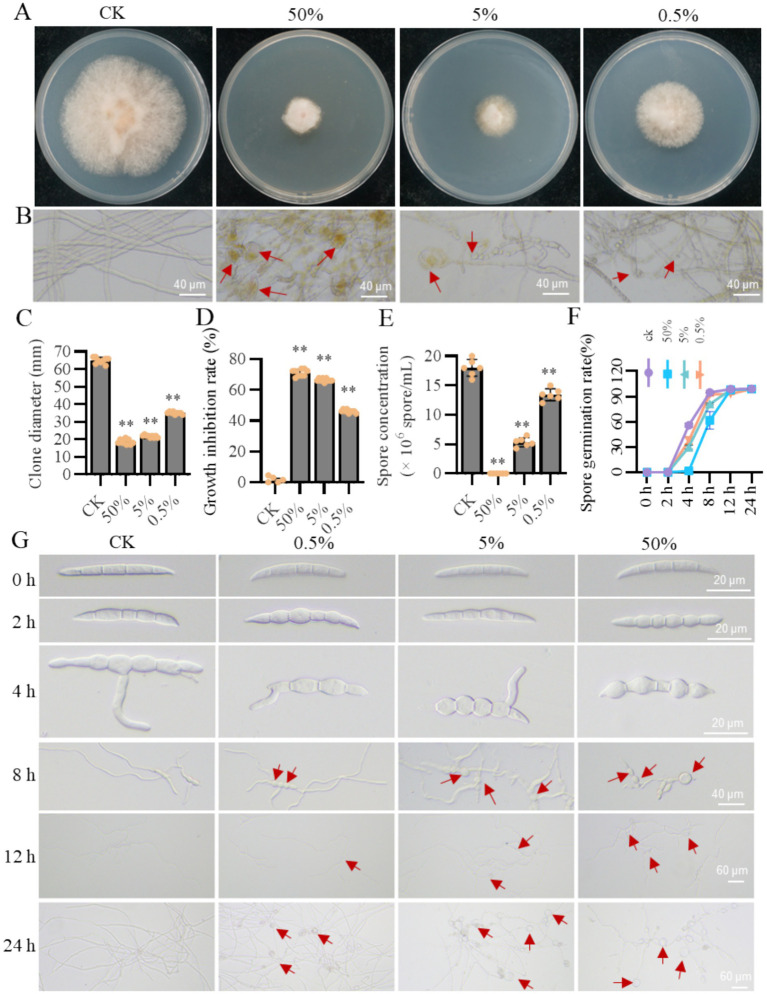
Analysis of antimicrobial activity of XH-10 fermentation broth. **(A)** Colony morphology of PH-1 cultured on plates containing different concentrations of XH-10 fermentation broth; **(B)** Microscopic morphology of hyphae at the colony margin of PH-1; **(C)** Quantitative analysis of PH-1 colony diameter; **(D)** Inhibition rate of PH-1 growth; **(E)** Statistical analysis of spore production in PH-1; **(F)** Germination rate statistics of PH-1 conidia in YEPD medium containing different concentrations of XH-10 fermentation broth; **(G)** Morphological observation of PH-1 spores and post-germination structures under different concentrations of XH-10 fermentation broth. Arrows indicate partially swollen structures; * indicates a significant difference vs. the control group (*p* < 0.05); ** indicates a highly significant difference (*p* < 0.01).

Analysis of the antagonistic activity of XH-10 fermentation broth revealed that, compared to the control, the mycelial growth of PH-1 was significantly inhibited upon addition of XH-10 fermentation broth. The inhibition rates were 71.11% (50% concentration), 66.49% (5% concentration), and 46.15% (0.5% concentration), with the inhibition rate increasing as the concentration of XH-10 increased (Figures 2A,C,D). These results indicate that XH-10 fermentation broth possesses antifungal activity against PH-1.

Microscopic observation revealed that upon addition of XH-10 fermentation broth, the mycelia of PH-1 exhibited swollen structures. When the concentration of XH-10 fermentation broth was increased, more pronounced mycelial swelling structures were observed (Figure 2B). In addition, spore production of PH-1 was reduced in the XH-10 fermentation broth treatment groups, with spore concentrations of 0 spore/mL (50% concentration), 5.33 × 10^6^ spore/mL (5% concentration), and 1.34 × 10^7^ spore/mL (0.5% concentration), compared to 1.80 × 10^7^ spore/mL in the control group, indicating that XH-10 fermentation broth inhibits spore production of PH-1 (Figure 2E).

Further analysis of the effect of XH-10 fermentation broth on conidial germination in *F. graminearum* HP-1 showed that the fermentation broth delayed this process. At 4 h of induction, the conidial germination rates of PH-1 were 55.81% in the control, and 1.78, 29.97, and 37.91% in 50, 5, and 0.5% XH-10 fermentation broth, respectively. As induction time progressed to 24 h, over 98% of PH-1 spores had germinated across all treatment groups, with rates of 99.05% in the control, and 98.90, 98.87, and 98.97% in 50, 5, and 0.5% XH-10 fermentation broth, respectively (Figure 2F).

Microscopic observation revealed that, compared to the control, conidia treated with XH-10 fermentation broth exhibited morphological changes, and the germinated mycelia displayed swollen structures to varying degrees. The higher the concentration of fermentation broth, the more pronounced the swollen structures (Figure 2G). In summary, the fermentation broth of strain XH-10 can inhibit mycelial growth and spore production of PH-1, delay conidial germination, and induce mycelial swelling and moniliform malformation, indicating that its metabolites are rich in effective antifungal active substances.

### 3.3 Effect of XH-10 on the pathogenicity of *F. graminearum*

**Figure 3:**
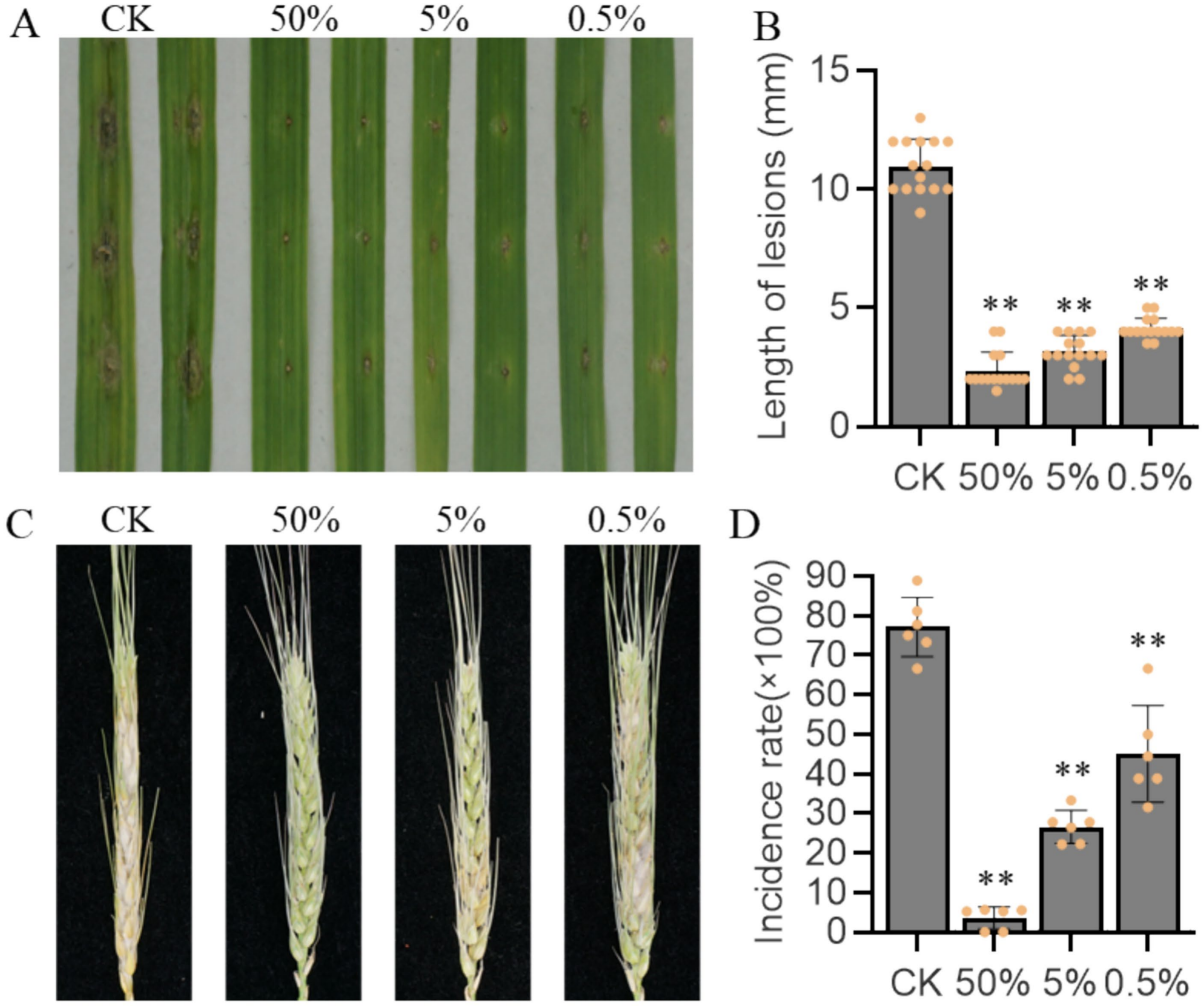
Pathogenicity analysis of XH-10 against PH-1. **(A)** Effect of XH-10 on PH-1 pathogenicity determined by indoor consultation inoculation method; **(B)** Statistical analysis of wheat leaf lesion length after indoor inoculation (*n* = 15); **(C)** Effect of XH-10 on PH-1 pathogenicity determined by single-flower inoculation method; **(D)** Statistical analysis of disease incidence rate in wheat ears after inoculation (*n* = 6). * Indicates a significant difference vs. the control group (*p* < 0.05); ** indicates a highly significant difference (*p* < 0.01).

To analyze the effect of XH-10 on the pathogenicity of *F. graminearum*, wound inoculation assays were performed on wheat leaves. The results showed that after inoculation with PH-1 combined with XH-10 fermentation broth, the lesions on wheat leaves were smaller compared to the control (Figures 3A,B). The inhibitory effect was stronger with increasing concentrations of XH-10, whereas wheat leaves inoculated with PH-1 alone exhibited severe disease symptoms.

Furthermore, single-floret inoculation assays on wheat spikes were conducted. The results were consistent with the leaf inoculation assays. The addition of XH-10 significantly weakened the pathogenicity of PH-1 in a concentration-dependent manner. Notably, when the concentration of XH-10 fermentation broth was 50%, the inhibitory effect on the pathogenicity of PH-1 was stronger (Figures 3C,D). These results indicate that the fermentation broth of XH-10 can inhibit the pathogenicity of *F. graminearum* PH-1strain.

### 3.4 Genome overview of strain XH-10

The complete genome sequence of XH-10 consists of a single circular chromosome, with a genome size of 4,099,941 bp and a GC content of 43.78%. Gene prediction revealed that strain XH-10 encodes a total of 4,233 genes, accounting for 89.73% of the total gene count, with a total coding sequence length of 3,678,941 bp and an average length of 869 bp. Among these, 4,027 CDS regions were identified, representing 88.13% of the total genes, with an average length of 897 bp. The genome contains 86 tRNA genes, 10 copies each of 23S rRNA, 16S rRNA, and 5S rRNA, one tmRNA, and 89 misc_RNA genes (Table 1). A total of 527 pseudogenes were identified, with a total length of 133,603 bp and an average length of 253.52 bp. Prediction of functional elements showed that strain XH-10 contains two CRISPR arrays, two genomic islands (17,781 bp and 100,569 bp in length, respectively), eight prophages, 198 repeat sequences, seven secondary metabolite biosynthetic gene clusters, one insertion sequence, and 5,076 promoter sequences.

**Table 1:** Statistics of strain XH-10 gene prediction results.

Type	Number	Total length	Average length	Percentage of genome (%)
Gene	4,233	3,678,941	869	89.73
CDS	4,027	3,613,104	897	88.13
tRNA	86	6,669	78	0.16
23S rRNA	10	29,250	2,925	0.71
16S rRNA	10	15,470	1,547	0.38
5S rRNA	10	1,110	111	0.03
tmRNA	1	360	360	0.01
misc_rna	89	12,978	146	0.32

Type: predicted gene type; Number: number of predicted genes; Total length: total length of predicted genes (bp); Average length: average gene length (bp); Percentage of genome (%): percentage of the total genome length.

**Figure 4:**
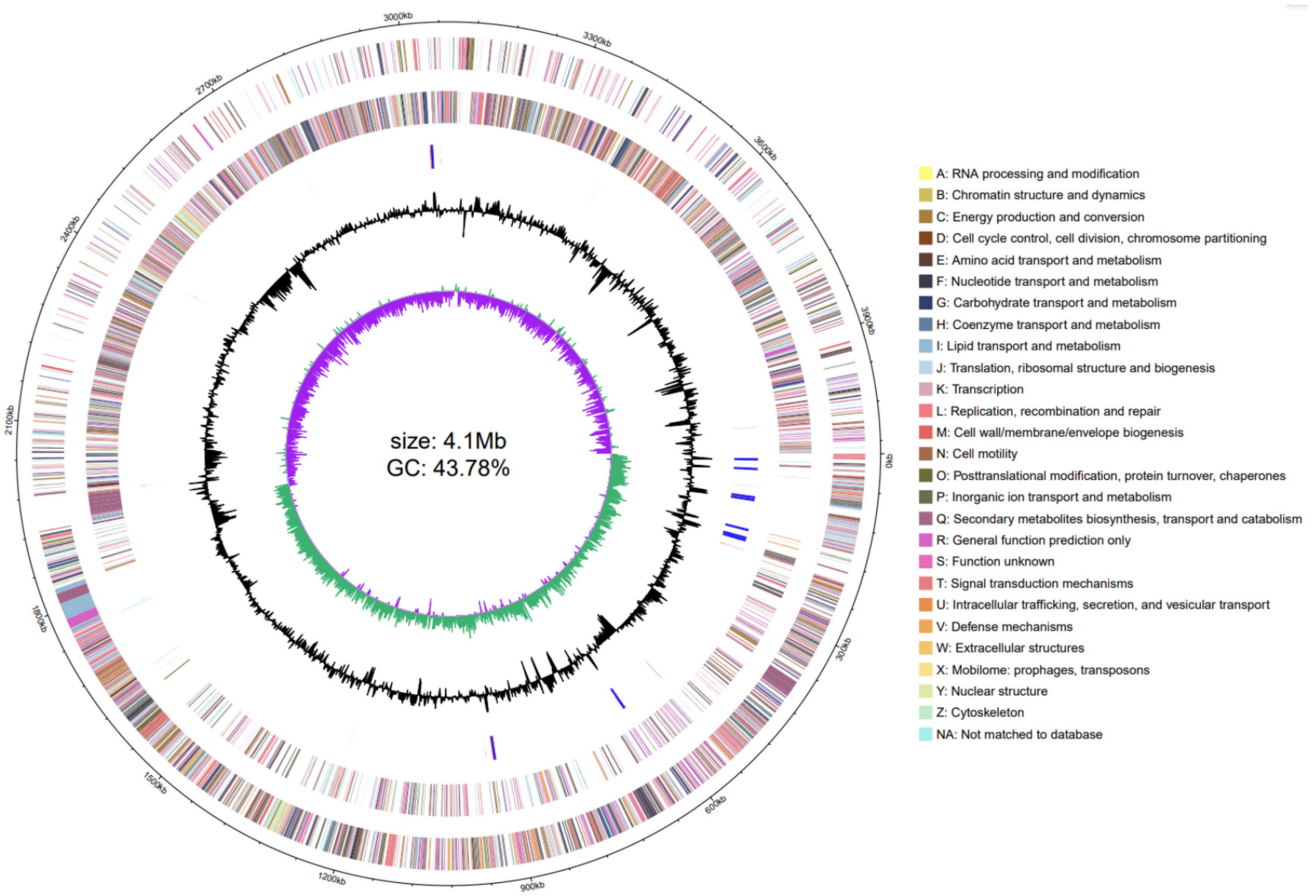
Genome circle plot of XH-10. The plot consists of six concentric circles from outer to inner. The outermost circle (1st) indicates genomic coordinates. The second and third circles represent genes on the positive and negative strands, respectively, with different colors denoting distinct COG functional categories. The fourth circle shows rRNA (blue) and tRNA (red). The fifth circle presents the average GC content calculated using a 2,000 bp sliding window. The sixth circle displays the GC skew (green for G > C, purple for G < C) with the same sliding window.

Using a multi-database combined annotation strategy, systematic functional annotation was performed on the 4,233 predicted coding genes in the genome of strain XH-10. The results showed that the NR database achieved the highest annotation efficiency, successfully matching 4,210 genes, covering 99.88% of all predicted genes. The RefSeq and UniProt databases followed closely, annotating 4,006 and 3,999 genes, with annotation rates of 99.48 and 99.30%, respectively. A total of 3,185 genes (78.10%) were annotated in the KEGG database. The COG and GO databases annotated 3,304 (78.39%) and 3,353 (79.55%) genes, respectively. The Pfam database annotated 3,593 genes, with a coverage rate of 86.50%, while the TIGRFAMs database annotated 2,298 genes, accounting for 54.52%. Based on the above genome structural annotation information, GC content distribution, and COG functional annotation results, the R package circlize was used for integrated visualization, generating a genome circle map of XH-10 (Figure 4). The annotation coverage rates across different databases exhibited a clear gradient distribution pattern: the NR, UniProt, and RefSeq databases showed relatively higher annotation efficiency, reflecting the high similarity between the gene sequences of strain XH-10 and known functional genes. In contrast, the annotation rate of the TIGRFAMs database was relatively low, which may be attributed to the fact that this database primarily annotates specific conserved domains, thus having certain limitations.

### 3.5 GO functional annotation of strain XH-10

**Figure 5:**
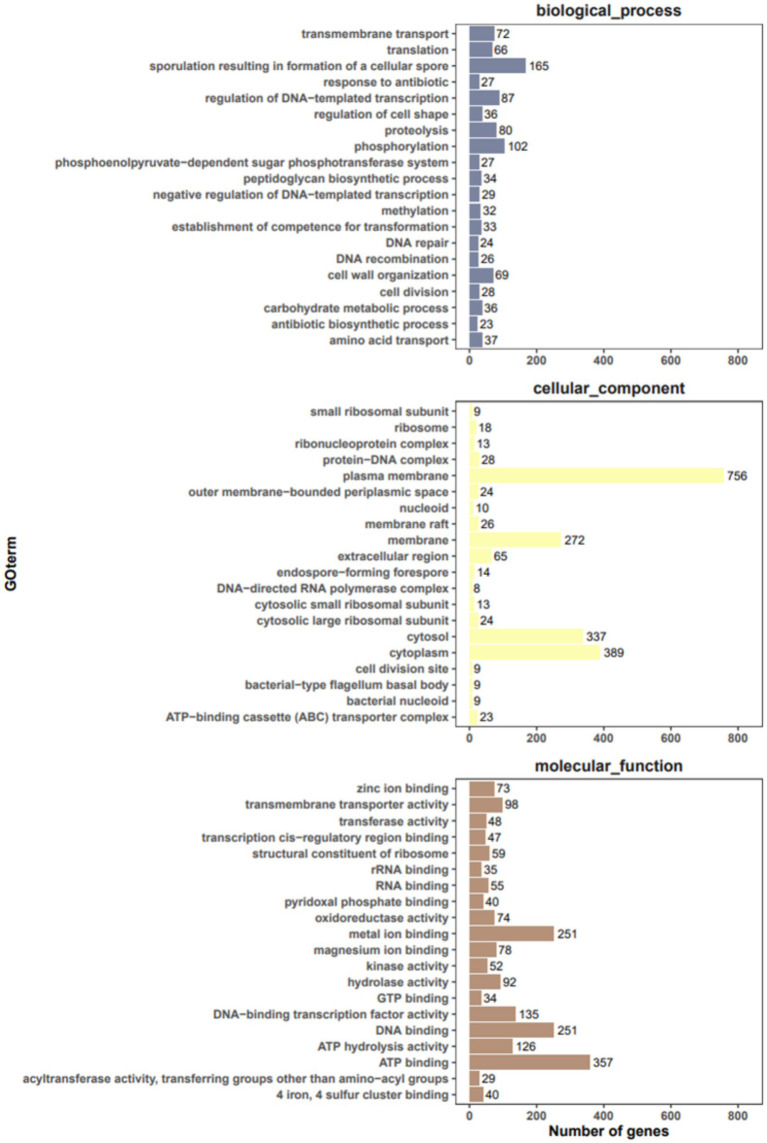
GO annotation result classification of XH-10. The *y*-axis represents the GO functional categories, and the *x*-axis represents the number of genes.

To systematically analyze the functional attributes of genes and their products in strain XH-10, the amino acid sequences were aligned and annotated against the Gene Ontology (GO) database. A total of 3,274 genes with GO annotations were obtained, distributed across the three main ontologies: Biological Process, Cellular Component, and Molecular Function (Figure 5).

In the Biological Process category, genes related to sporulation (sporulation resulting in formation of a cellular spore) showed the highest enrichment, with 165 genes, suggesting that strain XH-10 possesses a strong capacity for differentiation and dormant structure formation, which may be a key adaptive strategy for survival under adverse conditions and maintenance of population continuity. This was followed by regulation of DNA-templated transcription (87 genes), proteolysis (80 genes), cell wall organization (69 genes), transmembrane transport (72 genes), and phosphorylation (102 genes). Additionally, genes involved in translation (66 genes), DNA recombination (26 genes), DNA repair (24 genes), amino acid transport (37 genes), antibiotic biosynthesis (23 genes), carbohydrate metabolism (36 genes), cell division (28 genes), competence establishment (33 genes), methylation (32 genes), negative regulation of transcription (29 genes), peptidoglycan biosynthesis (34 genes), and the phosphoenolpyruvate-dependent sugar phosphotransferase system (27 genes) were also enriched. The widespread distribution of these genes indicates that XH-10 possesses a complete molecular basis for genetic information flow, transmembrane transport, basal metabolism, and environmental responses. Notably, the XH-10 genome contains genes involved in antibiotic biosynthesis, competence, and sporulation. This genetic repertoire suggests that the strain has the potential to adapt its physiology in response to changing environmental conditions.

In the Cellular Component category, plasma membrane-associated genes were the most abundant (756 genes), reflecting the central role of the plasma membrane as the cell-environment interface in material exchange and signal transduction. Basic structural components such as the cytoplasm (389 genes), cell membrane (272 genes), and cytosol (337 genes) were also enriched with a large number of genes, forming functional coupling with biological processes such as translation and metabolism. In addition, annotations involved specific components including the ATP-binding cassette transporter complex (23 genes), cytosolic large ribosomal subunit (24 genes), extracellular region (65 genes), outer membrane-bounded periplasmic space (24 genes), protein-DNA complex (28 genes), ribosome (18 genes), ribonucleoprotein complex (13 genes), membrane raft (26 genes), prospore (14 genes), and cytosolic small ribosomal subunit (13 genes). The presence of membrane raft-related genes is particularly noteworthy, as this structure has been reported as an assembly platform for signaling molecules and transporters; its enrichment further supports the functional advantage of XH-10 in signal perception and transmembrane transport. Meanwhile, the annotation of the prospore component provides subcellular localization evidence supporting the integrity of the sporulation process.

In the Molecular Function category, ATP binding-related genes were the most abundant (357 genes), highlighting the highly active energy metabolism of XH-10. This was followed by DNA binding (251 genes) and metal ion binding (251 genes), which ranked second with equal numbers, followed by transmembrane transporter activity (98 genes). Other prominent functions included ATP hydrolysis activity (126 genes), DNA-binding transcription factor activity (135 genes), GTP binding (34 genes), RNA binding (55 genes), hydrolase activity (92 genes), kinase activity (52 genes), magnesium ion binding (78 genes), and oxidoreductase activity (74 genes). This functional combination exhibits a clear hierarchical relationship: ATP binding and hydrolysis activity provide the energy driving force; DNA/RNA binding and transcription factor activity support the precise regulation of genetic information; while metal ion (especially magnesium ion) binding and oxidoreductase activity participate in the maintenance of enzyme catalysis and metabolic network homeostasis. Together, these three components form a molecular functional chain of “energy supply-information regulation-metabolic execution,” revealing at a deeper level the molecular logic by which XH-10 achieves efficient colonization and antagonistic functions in complex environments.

In summary, GO annotation analysis not only delineates the functional gene landscape of XH-10 at the macro level but also reveals, from micro perspectives such as sporulation, membrane raft structures, and functional molecular chains, the multiple molecular bases underlying this strain’s ability to adapt to the environment, compete for resources, and potentially antagonize pathogens, thereby providing more targeted research clues for the elucidation of its biocontrol mechanisms.

### 3.6 KEGG annotation of XH-10

**Figure 6:**
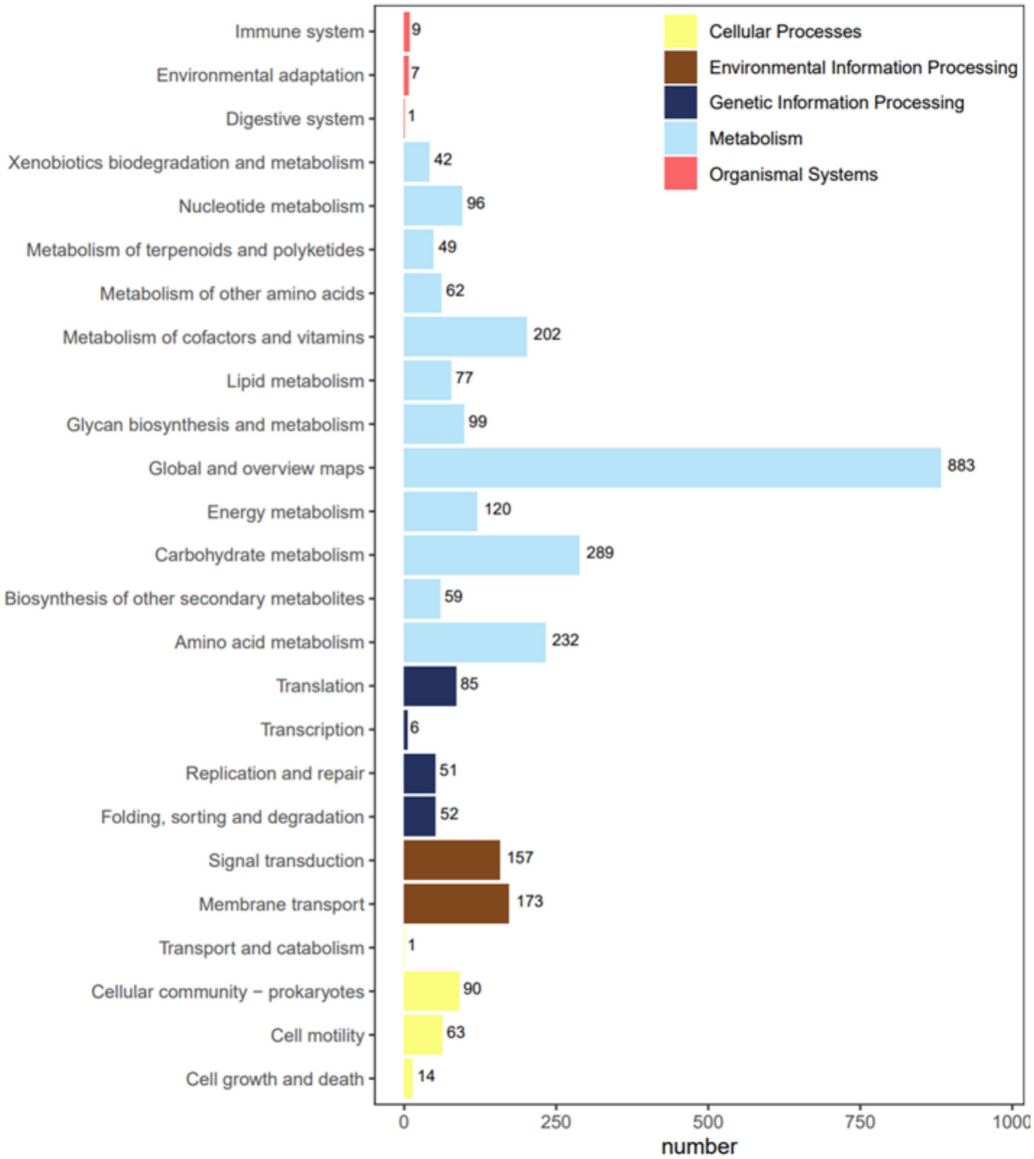
KEGG annotation plots of XH-10. The x-axis represents the number of annotated genes under each Pathway category, the *y*-axis represents the Pathway categories, and different colors indicate their respective major functional categories.

KEGG pathway annotation analysis was performed on strain XH-10, and the results showed that its functional genes were widely involved in various metabolic pathways and biological processes (Figure 6). Among the metabolic pathways, genes related to global metabolism overview were the most abundant, with 889 genes, indicating that XH-10 possesses a complete core metabolic network. In addition, genes involved in carbohydrate metabolism (230 genes) and amino acid metabolism (205 genes) were also relatively abundant. In terms of genetic information processing, genes involved in translation (84 genes), replication and repair (55 genes), and folding, sorting, and degradation (52 genes) were effectively annotated. In the environmental information processing pathways, genes related to membrane transport (175 genes) and signal transduction (152 genes) were particularly prominent. Furthermore, in the cellular processes and organismal systems pathways, genes involved in cell motility (63 genes) and the immune system (11 genes) were also annotated.

### 3.7 COG functional annotation of strain XH-10

**Figure 7:**
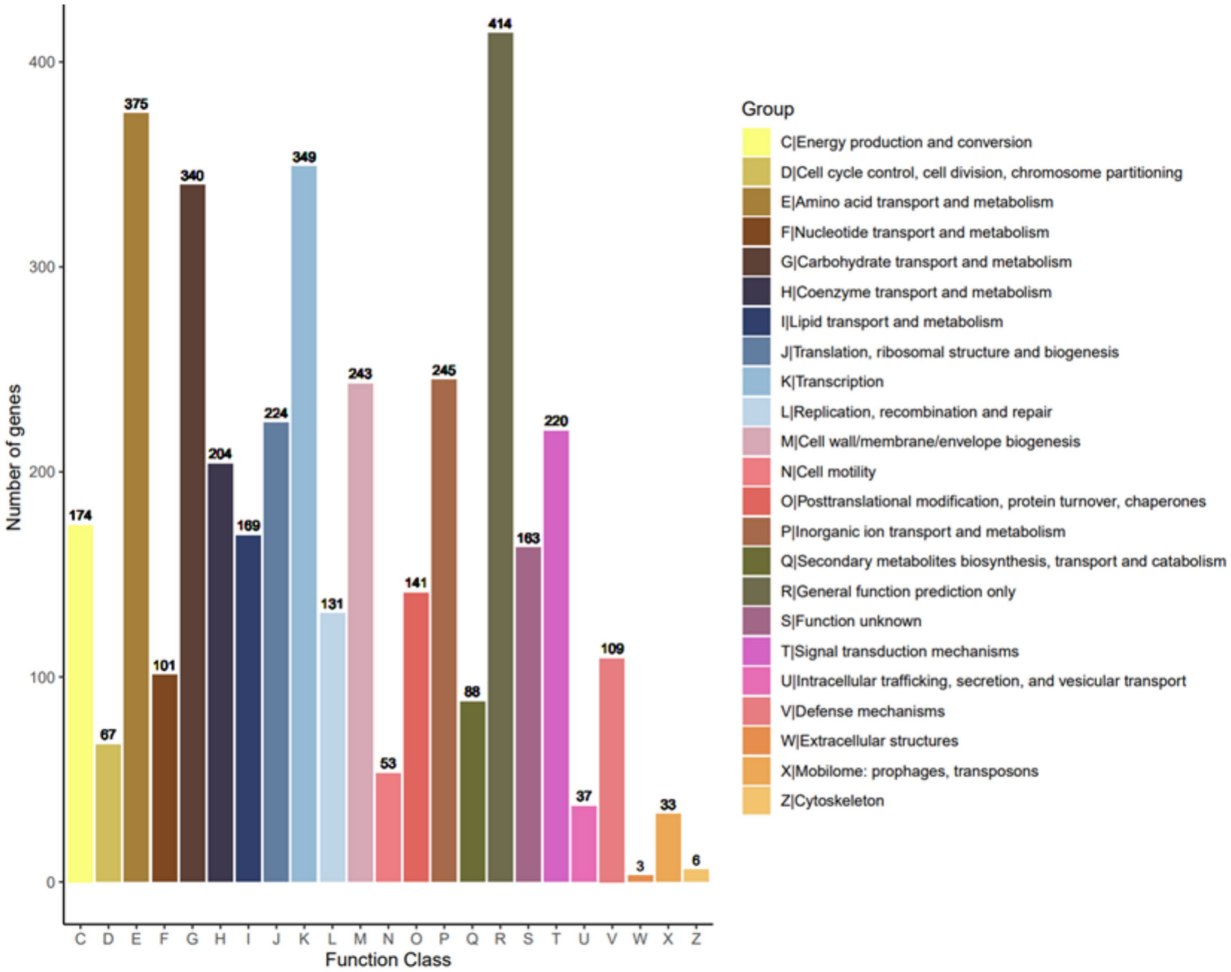
COG functional classification bar chart of the complete genome of strain XH-10. The *x*-axis represents the alphabetical codes of COG functional categories, and the legend on the right illustrates the biological function corresponding to each code. The *y*-axis shows the total number of genes annotated to each functional category.

Systematic analysis of the COG functional annotation results of strain XH-10 was performed to dissect the molecular basis of its metabolic characteristics and biocontrol potential. The results showed that a total of 88 genes were annotated to the category of secondary metabolite biosynthesis, transport, and catabolism (Class Q) (Figure 7), which represents a relatively high number among all functional categories. The enrichment of Class Q genes suggests that XH-10 possesses a strong potential for secondary metabolite synthesis, laying the foundation for its antagonistic activity against *F. graminearum*.

At the level of basal metabolism, there were 375 genes related to amino acid metabolism (Class E) and 340 genes related to carbohydrate metabolism (Class G), which together accounted for the highest proportion among all functional genes. In addition, 169 genes were annotated to lipid transport and metabolism (Class I), 204 genes to coenzyme transport and metabolism (Class H), 101 genes to nucleotide transport and metabolism (Class F), and 245 genes to inorganic ion transport and metabolism (Class P). The distribution of these metabolism-related genes indicates that this strain possesses efficient capabilities for carbon, nitrogen, lipid, coenzyme, nucleotide, and ion metabolism, which favor its rapid proliferation and ecological niche dominance in complex environments. Meanwhile, 174 genes were annotated to energy metabolism (Class C), 67 genes to cell cycle control, cell division, and chromosome partitioning (Class D), and 53 genes to cell motility (Class N). Together, these gene modules constitute the fundamental system for energy supply, cell division, and motility in this strain.

At the level of genetic information processing, 45 genes were annotated to translation (Class J), 98 genes to transcription (Class K), and 131 genes to replication, recombination, and repair (Class L). These core functional modules collectively ensure the stable transmission and expression of genetic information, supporting the normal physiological activities of the strain under stress conditions.

At the level of environmental adaptation and regulation, 220 genes were annotated to signal transduction (Class T) and 109 genes to defense mechanisms (Class V), a distribution similar to that of biocontrol strains such as *B. subtilis* DNKAS. The abundant signal transduction genes endow XH-10 with the ability to rapidly perceive changes in the external environment, while defense-related genes provide protection against adverse environmental factors. Notably, 243 genes were annotated to cell membrane biogenesis (Class M), and their high abundance is closely related to the strong biofilm-forming capacity of this strain. Biofilm formation not only facilitates the colonization of the strain on wheat spike surfaces but also reduces the chances of pathogen infection through physical barriers.

In the overall composition of functional genes, 414 genes were classified into general function prediction only (Class R), and 163 genes were classified into function unknown (Class S). The abundance of these two categories occupies a significant proportion of the COG classification, reflecting the presence of a large number of sequences whose functions have not yet been elucidated or can only be predicted in the XH-10 genome.

From the perspective of overall functional gene distribution, the COG annotation profile of XH-10 exhibits characteristics of “dominance by metabolism-related genes, abundance of defense-related genes, completeness of regulatory genes, and a relatively high proportion of genes with unknown function.” This functional architecture is highly consistent with the metabolic characteristics of *B. subtilis* as a typical biocontrol agent, indicating that this strain possesses comprehensive advantages in nutrient competition, environmental adaptation, and antimicrobial synthesis. In summary, the COG annotation results reveal the biocontrol potential of XH-10 at the functional gene level and provide important clues for subsequent molecular mechanism studies.

### 3.8 CAZyme annotation of XH-10

CAZyme annotation analysis of the strain XH-10 genome was performed using the CAZy database. A total of 138 coding genes were identified, distributed across six major CAZyme families (Table 2). Among these, the glycoside hydrolase family (GHs) contained the largest number of genes (59), followed by the glycosyltransferase family (GTs, 41 genes), carbohydrate esterase family (CEs, 17 genes), carbohydrate-binding module family (CBMs, 9 genes), polysaccharide lyase family (PLs, 7 genes), and auxiliary activity family (AAs, 5 genes). GHs and GTs were the dominant families, accounting for 42.75 and 29.71% of the total annotated genes, respectively.

**Table 2:** CAZyme result of strain XH-10 genome.

Carbohydrate-active enzyme family	Number of genes	Gene subfamily(number of genes)
Glycoside hydrolases, GHs	59	GH1(5), GH105(2), GH11(1), GH126(1), GH13(8), GH16(1), GH171(1), GH177(5), GH179(3), GH18(4), GH23(1), GH26(2), GH3(1), GH30(1), GH32(3), GH36(1), GH4(4), GH42(2), GH43(4), GH46(1), GH5(1), GH51(2), GH53(1), GH65(1), GH68(1), GH73(2)
Glycosyl transferases, GTs	41	GT1(3), GT2(18), GT26(1), GT28(2), GT35(1), GT4(8), GT5(1), GT51(4), GT8(1), GT83(2)
Carbohydrate esterases, CEs	17	CE1(3), CE12(3), CE14(2), CE19(1), CE4(6), CE7(1), CE9(1)
Carbohydrate-binding modules, CBMs	9	CBM23(1), CBM26(1), CBM3(1), CBM34(1), CBM6(1), CBM63(1), CBM66(1), CBM68(1), CBM91(1)
Polysaccharide lyases, PLs	7	PL1(2), PL11(2), PL26(1), PL3(1), PL9(1)
Auxiliary activities, AAs	5	AA1(1), AA4(1), AA6(1), AA7(2)

At the functional subfamily level, GH18, GH23, and CBM50 have been reported to be involved in the encoding of chitinases, suggesting that XH-10 has the potential to degrade chitin and related complexes. GH1 and GH9 are involved in the catabolism of cellulose and hemicellulose, while GH126 specifically encodes starch hydrolases. The combined configuration of these enzymes indicates that strain XH-10 not only can utilize monosaccharides or oligosaccharide substrates but also possesses the direct capacity to degrade macromolecular polysaccharides such as chitin, cellulose, hemicellulose, and starch. This broad substrate spectrum provides a molecular basis for the utilization of diverse carbon sources in plant rhizosphere or spike environments and may also indirectly exert antagonistic effects by degrading components of the pathogenic fungal cell wall, such as chitin.

### 3.9 Secondary metabolic potential of strain XH-10

**Figure 8:**
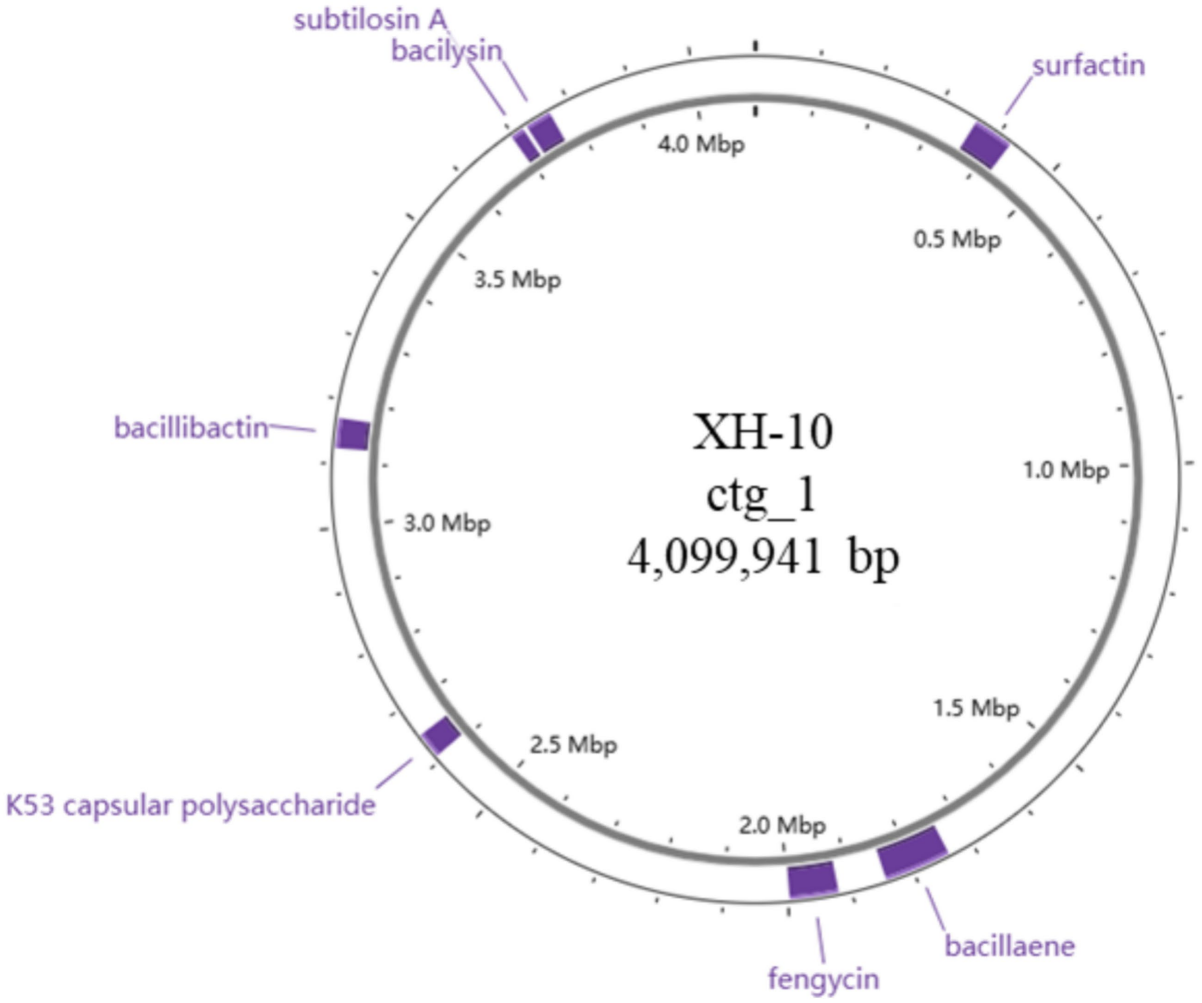
Gene cluster map of secondary metabolites of XH-10. The outermost and inner circles indicate the genomic coordinates (in Mbp). The seven purple arcs on the circles indicate the positions of the seven predicted gene clusters on the genome, which are responsible for the biosynthesis of surfactin (a lipopeptide with surfactant and antimicrobial activity), bacillaene (a polyene antibiotic), fengycin (an antifungal lipopeptide), K53 capsular polysaccharide (involved in polysaccharide biosynthesis), bacillibactin (a siderophore for iron acquisition), subtilosin A (an antimicrobial peptide), and bacilysin (a dipeptide antibiotic), respectively.

Genome mining analysis using antiSMASH identified a total of seven secondary metabolite biosynthetic gene clusters in strain XH-10, involving various biosynthesis mechanisms such as non-ribosomal peptide synthetase (NRPS), polyketide synthase (PKS), and ribosomally synthesized and post-translationally modified peptides (RiPPs) (Figure 8). The metabolites encoded by these gene clusters mainly include surfactin, bacillaene, fengycin, K53 capsular polysaccharide, bacillibactin, subtilosin A, and bacilysin.

From the perspective of biosynthesis pathways, surfactin and bacillibactin are typical NRPS types; fengycin possesses both betalactone and NRPS features; bacillaene integrates multiple pathways including PKS-like, T3PKS, NRPS, and transAT-PKS; K53 capsular polysaccharide is synthesized via an NRPS-like pathway; subtilosin A belongs to the sactipeptide class of RiPPs; and bacilysin is a non-ribosomally synthesized dipeptide (classified as “other” by antiSMASH). Most of these metabolites have been reported to exhibit antibacterial or antagonistic functions in *Bacillus* species. Therefore, it is speculated that strain XH-10 may exert antagonistic activity against *F. graminearum* through the synthesis of these active substances (Table 3).

**Table 3:** Prediction statistics of Biosynthetic Gene Clusters (BGCs).

**Compound**	**Synthetase Type**	**Genes**	**Size(Kb)**
surfactin	NRPS	*nasC*, *nasB*, *yciC*, *yckB*, *srfAA*, *srfAB*, *srfAC*, *srfAD*, *gabR_1*, *sfp, tcyA*, *bsdB*, *pip_2*	63.4
bacillaene	PKS-like, T3PKS, NRPS, transAT-PKS	*pbpX*, *tdh*, *miaB*, *pksB*, *pksC*, *pksD*, *pksE*, *acpK*, *pksF*, *pksG*, *pksH*, *pksI*, *pksJ*, *pksL*, *pksM*, *pksN*, *pksR*, *pksS_1*, *aprX*	105.3
fengycin	betalactone, NRPS	*gtaB_1*, *accD5*, *crt*, *yngG*, *cfiB*, *fadD3*, *bcd*, *ppsE*, *ppsD*, *ppsC*, *ppsB*, *ppsA*, *ggt*, *adhT*	76.9
K53 capsular polysaccharide	NRPS-like	*cycA_2*, *ylmB_3*, *carB_3*, *garR_2*, *grsB*, *wbgU_1*, *wbpA*, *cypB*, *yrhH*, *mccB*, *mccA*	41.2
bacillibactin	NRPS	*rutB_2*, *yueD*, *mbtH*, *dhbF*, *dhbB*, *dhbE*, *dhbC*, *dhbA*, *besA*, *yumC*	47.1
subtilosin A	sactipeptide	*narG*, *sboA*, *albA*, *albE*, *albF*	21.6
bacilysin	other	*bacG*, *bacF_2*, *bacD*, *bacC_1*, *rocC_2*, *rocA, rfbC*, *rmlD*, *rfbB*, *rmlA*, *murG_2*, *arnB*	41.4

## 4 Discussion

Fusarium head blight (FHB) is one of the most destructive wheat diseases worldwide, posing a serious threat to global food security. Currently, the management of this disease relies primarily on chemical fungicides. However, long-term application of single fungicides has led to the emergence of resistance in field populations of *F. graminearum*, accompanied by issues such as pesticide residues and environmental pollution (Naqvi et al., 2025). In recent years, significant progress has been made in the use of *B. subtilis* and its secondary metabolites for the control of plant diseases. Due to its broad-spectrum antimicrobial activity, strong stress tolerance, and high safety profile, *B. subtilis* has become an important microbial resource for the development of biological pesticides (Zhu et al., 2026; Cheng et al., 2025). In this study, XH-10 was isolated from farmland soil. Through *in vitro* antagonistic assays, pathogenicity tests, and whole-genome sequencing, the antagonistic phenotype of this strain against PH-1 was characterized, and the potential biocontrol genetic basis was elucidated at the genomic level. This study aims to provide theoretical references for the mining of biocontrol resources against FHB and the elucidation of their mechanisms.

Previous studies have shown that biocontrol bacteria primarily exert their effects by secreting extracellular antimicrobial substances that disrupt the cell wall and cell membrane structures of pathogens, damage hyphae and spores, cause protoplast leakage and hyphal disintegration, and inhibit conidial germination and sporulation while inducing spore malformation (Yu et al., 2021). Studies on *B. subtilis* have further confirmed this mechanism: strain KB-1122 secretes *β*-glucanase to degrade pathogen cell wall polysaccharides, thereby achieving bacteriolytic effects (Zhang et al., 2009); strains SYX20 and SYX04 simultaneously secrete chitinase and β-1,3-glucanase, and the synergistic action of these two enzymes decomposes the cell walls of hyphae and conidia, inducing protoplast exosmosis (Sha et al., 2016). The results of the present study are consistent with the above findings. *In vitro* antagonistic assays demonstrated that the fermentation broth of XH-10 significantly inhibited the growth and development of PH-1 in a concentration-dependent manner. The inhibition rates of mycelial growth at fermentation broth concentrations of 50, 5, and 0.5% were 71.11, 66.49, and 46.15%, respectively, and the fermentation broth caused mycelial swelling and moniliform malformation, with more pronounced malformation phenotypes at higher concentrations. Meanwhile, the XH-10 fermentation broth effectively inhibited sporulation and conidial germination of *F. graminearum*. Under high-concentration conditions, sporulation was completely inhibited, demonstrating an antagonistic effect superior to most similar biocontrol strains. Given that spore formation and germination are key steps in the field transmission, spread, and re-infection of FHB, the potent inhibition of pathogen sporulation by XH-10 suggests that this strain possesses promising potential for field disease management. Based on these findings, it is speculated that XH-10 secretes one or more extracellular antimicrobial active substances that exert antifungal effects by disrupting fungal cell structures and hindering their growth and development. Although this study clarified the overall antifungal function of the XH-10 fermentation broth, the core active components and their specific mechanisms of action remain unclear. Future studies involving the isolation and purification of active substances are needed to further elucidate the regulatory mechanisms by which XH-10 affects the growth and development of *F. graminearum*.

The whole-genome sequencing results provide a reliable genetic background for elucidating the biocontrol potential of XH-10. The genome size, GC content, and gene number of this strain are generally consistent with those of typical reference strains of *B. subtilis* (Zeigler et al., 2008; Schroeder and Simmons, 2013), indicating that its genome structure is conserved and its genetic background is stable. Genome mining predicted a total of seven secondary metabolite biosynthetic gene clusters, encompassing biosynthetic pathways for various typical antimicrobial substances, including surfactin, fengycin, and bacillibactin. This is highly similar to the secondary metabolic genetic background of highly efficient biocontrol strains such as SNBS-3 (Li et al., 2020) and TCS001 (Jiang et al., 2025), suggesting that such gene clusters constitute a conserved genetic basis for the antagonistic activity of *B. subtilis* against filamentous fungi. Meanwhile, COG annotation revealed an enrichment of genes related to secondary metabolite biosynthesis and transport (Class Q), further indicating that XH-10 possesses the genetic potential to synthesize diverse antimicrobial active substances. Compared with most biocontrol strains that harbor only 2–5 major gene clusters, XH-10 exhibits a richer diversity of secondary metabolite gene clusters, suggesting a potential mode of multi-pathway, multi-component synergistic antimicrobial activity that could circumvent the limitations of a single antimicrobial mechanism. However, the synergistic regulatory mechanisms involved require further experimental validation.

Iron competition represents a classic potential mechanism by which biocontrol bacteria participate in microecological niche competition and inhibit pathogen infection. Studies have shown that in the rhizosphere environment where bioavailable iron is extremely limited, biocontrol bacteria can effectively inhibit pathogen growth and infection by secreting siderophores to compete for environmental iron ions (Shao et al., 2024). Siderophore-mediated iron competition not only directly affects the physiological metabolism of pathogens but also enhances the overall disease-suppressive function of the rhizosphere microbiome by regulating intercellular interactions within the microbial community (Shao et al., 2024). Genome sequence analysis in this study revealed that XH-10 contains genes related to siderophore biosynthesis (*dhbA*, *dhbB*, *dhbC*, *dhbE*, *dhbF*), providing the genetic basis for the synthesis of bacillibactin. Previous studies have confirmed that siderophores produced by *B. subtilis* possess a strong iron-chelating capacity, which can inhibit pathogen growth and infection by depriving them of limited environmental iron sources (Lyng et al., 2024; Ortiz et al., 2026). Therefore, it is speculated that iron competition is a potential pathway by which XH-10 assists in inhibiting *F. graminearum*. Compared with strains that rely solely on a single mechanism of metabolite-based antimicrobial activity, XH-10 possesses, at the genomic level, two functional systems: antimicrobial metabolite synthesis and iron nutrient competition (the latter mediated by the *dhbA*-*dhbF* gene cluster encoding bacillibactin). This suggests that the biocontrol mechanism of XH-10 is more diverse; however, the actual function of this mechanism requires further validation.

CAZyme functional annotation results revealed the genetic potential of XH-10 for both disease suppression and environmental adaptation. This strain contains abundant glycoside hydrolase genes, among which the GH18, GH23, and CBM50 family genes can encode chitinases, providing the genetic basis for degrading core components of the *F. graminearum* cell wall. This is consistent with the cell wall degradation mechanisms of most biocontrol *Bacillus* strains (Ajuna et al., 2023; Riseh et al., 2024). Meanwhile, the strain contains genes encoding plant polysaccharide-degrading enzymes such as GH5 and GH43, which could support its utilization of wheat surface polysaccharides as carbon sources. Studies have shown that GH5 (endoglucanase) and GH43 (xylanase) from *Bacillus* sources are responsible for the efficient decomposition of cellulose and hemicellulose components in plant polysaccharides (Srivastava et al., 2022; Yang et al., 2025). Combined with the significant enrichment of carbohydrate metabolism and amino acid metabolism pathways in the KEGG annotation, these findings suggest that XH-10 possesses a metabolic regulatory basis adapted to the wheat spike microenvironment. By efficiently utilizing environmental nutrients, it can enhance its colonization competitiveness and thereby indirectly exert biocontrol effects (Chen et al., 2024). These results explain, at the genomic level, the dual genetic advantages of this strain in both antifungal activity and environmental adaptation. The significant enrichment of sporulation-related genes reveals the outstanding field application potential and stress resistance adaptability of XH-10.

GO annotation results showed that sporulation-related genes constituted the most highly enriched functional gene category in this strain, indicating that spore formation is one of its core physiological characteristics (de Hoon et al., 2010). From an application perspective, the high stress resistance of spores can address the industrial challenges of low field survival rates and poor storage stability of many biocontrol agents. From an ecological perspective, the efficient sporulation capacity enables the strain to survive in a dormant form under adverse conditions such as the non-growth period of wheat, high temperature and drought, and chemical stress, and then rapidly resuscitate and colonize when environmental conditions become favorable, achieving continuous disease control across growth seasons (Mulyani et al., 2025). Furthermore, based on studies of similar strains, the presence of environmental tolerance genes such as *clpP/C* (ATP-dependent protease, not shown in the results section) in XH-10 suggests that these genes may help XH-10 remove misfolded proteins under environmental stress (e.g., pesticide residues, extreme temperatures), maintain cellular homeostasis, and ensure the stability of biocontrol functions. Therefore, they may enhance the adaptability of the strain in fermentation production and field environments.

In summary, through antagonistic assays, antifungal tests, and whole-genome sequencing analysis, this study has preliminarily characterized the biocontrol potential of XH-10. At the genomic level, this strain possesses multiple functional gene modules, including those for secondary metabolite synthesis, cell wall degradation, nutrient competition, and stress-resistant survival. These genetic features may contribute to its antagonistic activity, potentially through cooperative actions among these modules. However, it should be noted that these findings are currently based on genomic predictions and *in vitro* phenotypic assays. Further transcriptomic or metabolomic analyses are required to verify the actual expression and functional coordination of these gene modules *in vivo*. The antifungal phenotype and genetic makeup of XH-10 suggest advantages over some previously reported strains, yet comprehensive comparisons under standardized conditions are needed to fully evaluate its biocontrol potential.

All mechanistic analyses in this study are based on *in vitro* laboratory experiments and genomic bioinformatics predictions. The core gene functions, key metabolic active components, field colonization capacity, and actual control efficacy of the strain have not yet been validated, representing certain limitations of this study. Future studies involving gene expression validation, isolation and purification of active substances, and field trials are needed to further elucidate the biocontrol mechanisms of XH-10, providing a more solid theoretical basis for the industrialization of this strain and the green control of FHB.

## 5 Conclusion

A bacterial strain XH-10 with strong antagonistic activity against *F. graminearum* PH-1, the major causal agent of wheat *Fusarium* head blight (FHB), was isolated from soil and identified as *Bacillus subtilis* based on morphological characteristics, Gram staining, and 16S rDNA analysis. The fermentation broth of XH-10 inhibited PH-1 in a concentration-dependent manner, with an inhibition rate of 71.11% at 50% concentration. It also induced hyphal malformations (swelling and moniliform enlargement), suppressed conidial germination and sporulation, and reduced PH-1 infectivity toward wheat leaves and spikes. Whole-genome sequencing revealed a genome of 4,099,941 bp encoding 4,233 genes, containing CAZyme gene clusters and seven secondary metabolite biosynthetic gene clusters (e.g., surfactin, fengycin, and bacillibactin). This study provides a novel microbial resource and genomic basis for developing biocontrol agents against wheat FHB.

## Data Availability

The raw genome sequencing data have been deposited in the Genome Warehouse of the National Genomics Data Center (NGDC) under accession number GWHJMXG01000000, and are publicly accessible at https://ngdc.cncb.ac.cn/gwh.

## Footnotes

1
https://www.megasoftware.net/

2
https://github.com/ctSkennerton/minced

3
https://isfinder.biotoul.fr/

4
https://dna.mbu.iisc.ac.in/prompredict/prompredict-intro.html

5
https://www.kegg.jp/kegg/

6
http://geneontology.org/

7
https://pfam.xfam.org/

8
https://www.ncbi.nlm.nih.gov/research/COG

9
http://tigrfams.jcvi.org/cgi-bin/index.cgi

10
https://ftp.ncbi.nlm.nih.gov/blast/db/FASTA/

11
https://blast.ncbi.nlm.nih.gov/Blast.cgi

12
https://www.ibm.com/spss

## References

[ref1] Ajuna H. B. Lim H. I. Moon J. H. Won S. J. Choub V. Choi S. I. . (2023). The prospect of hydrolytic enzymes from *Bacillus* species in the biological control of pests and diseases in forest and fruit tree production. Int. J. Mol. Sci. 24:16889. doi: 10.3390/ijms242316889, 38069212 PMC10707167

[ref2] Akhter S. Aziz R. Edwards R. (2012). PhiSpy: a novel algorithm for finding prophages in bacterial genomes that combines similarity-and composition-based strategies. Nucleic Acids Res. 40, e126–e126. doi: 10.1093/nar/gks406, 22584627 PMC3439882

[ref3] Amjad B. Sultan S. Abro A. Tabassum B. Ilyas M. Jahan N. . (2026). Whole-genome sequencing and biosynthetic gene cluster analysis of *Bacillus subtilis* BAGL as a potent antifungal biocontrol agent against phytopathogenic fungi. Int. Microbiol. 29, 549–564. doi: 10.1007/s10123-026-00802-7, 41820735 PMC13083322

[ref4] Audenaert K. Callewaert E. Höfte M. De Saeger S. Haesaert G. (2010). Hydrogen peroxide induced by the fungicide prothioconazole triggers deoxynivalenol (DON) production by *fusarium graminearum*. BMC Microbiol. 10:112. doi: 10.1186/1471-2180-10-112, 20398299 PMC2859870

[ref5] Blin K. Shaw S. Kloosterman A. Charlop-Powers Z. Wezel G. Medema M. . (2021). AntiSMASH 6.0: improving cluster detection and comparison capabilities. Nucleic Acids Res. 49, W29–W35. doi: 10.1093/nar/gkab335 33978755 PMC8262755

[ref6] Chen X. Zhang Y. Chao S. Song L. Wu G. Sun Y. . (2024). Biocontrol potential of endophytic *Bacillus subtilis* A9 against rot disease of *Morchella esculenta*. Front. Microbiol. 15:1388669. doi: 10.3389/fmicb.2024.1388669, 38873148 PMC11169702

[ref7] Cheng W. Zhou L. Jiang C. Zhao C. Song J. Wang Q. . (2025). *Bacillus subtilis* 0618A volatiles inhibit sclerotium rolfsii and enhance soil health in the biocontrol of peanut southern blight. J. Agric. Food Chem. 73, 22321–22332. doi: 10.1021/acs.jafc.5c09410, 40887889

[ref8] Cho Y. T. Ting H. M. Wang B. W. Tsai Y. C. Wang H. Y. Yang Y. L. . (2025). Integrating genomics and targeted metabolite profiling to elucidate disease-suppression mechanisms of *Bacillus velezensis* GFB08. Curr Res Microb Sci. 9:100503. doi: 10.1016/j.crmicr.2025.100503, 41281783 PMC12637390

[ref9] de Hoon M. J. Eichenberger P. Vitkup D. (2010). Hierarchical evolution of the bacterial sporulation network. Curr. Biol. 20, R735–R745. doi: 10.1016/j.cub.2010.06.031, 20833318 PMC2944226

[ref10] Duncan D. B. (1955). Multiple range and multiple f tests. Biometrics 11, 1–42. doi: 10.2307/3001478

[ref11] Finn R. D. Clements J. Eddy S. R. (2011). HMMER web server: interactive sequence similarity searching. Nucleic Acids Res. 39, W29–W37. doi: 10.1093/nar/gkr367, 21593126 PMC3125773

[ref12] Horo J. T. He X. Danu K. G. Hei N. B. Megerssa S. H. Zhang X. (2026). Fusarium head blight: emergence, impacts on wheat production and management in Ethiopia and beyond. Front. Agron. 7:1716770. doi: 10.3389/fagro.2025.1716770

[ref13] Hsiao W. Wan I. Jones S. Brinkman F. (2003). IslandPath: aiding detection of genomic islands in prokaryotes. Bioinformatics 19, 418–420. doi: 10.1093/bioinformatics/btg004, 12584130

[ref14] Jiang H. Z. Liu L. Y. Yang R. D. Chen J. Jin J. (2025). Genome mining and metabolite profiling uncover the biocontrol and plant growth-promoting capacities of *Bacillus velezensis* TCS001. Biol. Control 211:105928. doi: 10.1016/j.biocontrol.2025.105928

[ref15] Kashyap P. L. Kumar S. Khanna A. Jasrotia P. Singh G. (2025). Sustainable microbial solutions for managing fungal threats in wheat: progress and future directions. World J. Microbiol. Biotechnol. 41:79. doi: 10.1007/s11274-025-04286-x, 40011267

[ref16] Li J. Gu T. Li L. Wu X. Shen L. Yu R. . (2020). Complete genome sequencing and comparative genomic analyses of *Bacillus sp.* S3, a novel hyper Sb(III)-oxidizing bacterium. BMC Microbiol. 20:106. doi: 10.1186/s12866-020-01737-3, 32354325 PMC7193398

[ref17] Liao Y. Xu X. Peng H. Chen A. Hao C. Li C. (2026). Biocontrol efficacy of *Bacillus velezensis* Fxj against *Fusarium graminearum*-induced *fusarium* head blight in wheat. J Fungi (Basel). 12:37. doi: 10.3390/jof12010037, 41590449 PMC12842974

[ref18] Liu E. Chen Y. Yao Y. Pei W. Pu T. Shi Z. . (2026). Whole genome analysis of *Bacillus velezensis* YNK-FB0059 and its multifunctional plant growth-promoting and biocontrol potential. Front. Microbiol. 17:1748090. doi: 10.3389/fmicb.2026.1748090, 41695943 PMC12901434

[ref19] Lyng M. Jørgensen J. P. B. Schostag M. D. Jarmusch S. A. Aguilar D. K. C. Lozano-Andrade C. N. . (2024). Competition for iron shapes metabolic antagonism between *Bacillus subtilis* and *Pseudomonas marginalis*. ISME J. 18:wrad001. doi: 10.1093/ismejo/wrad001, 38365234 PMC10811728

[ref20] Mourelos C. A. Malbrán I. Mengual Gómez D. Ghiringhelli P. D. Lori G. A. (2024). Dynamics of *fusarium graminearum* inoculum on residues of naturally infected winter and summer crops. Eur. J. Plant Pathol. 169, 543–553. doi: 10.1007/s10658-024-02850-z

[ref21] Mulyani Y. Ilyas S. Machmud M. (2025). Development of biocontrol formula and its effectiveness for seed treatment in managing bacterial leaf blight and improving seed quality and yield of rice. IOP Conf. Ser. Earth Environ. Sci. 1528:012028. doi: 10.1088/1755-1315/1528/1/012028

[ref22] Naqvi S. A. H. Farhan M. Ahmad M. Kiran R. Shahbaz M. Abbas A. . (2025). Fungicide resistance in *fusarium* species: exploring environmental impacts and sustainable management strategies. Arch. Microbiol. 207:31. doi: 10.1007/s00203-024-04219-6, 39792175

[ref23] Naser S. R. Chowdhury S. F. Sarkar M. M. H. Habib M. A. Akter S. Banu T. A. . (2025). Exploring the potential of *Bacillus spp.* for secondary metabolite production through genome mining approaches. J. Genet. Engin. Biotechnol. 23:100595. doi: 10.1016/j.jgeb.2025.100595, 41386860 PMC12642121

[ref24] Nikolaeva A. Pudova D. S. Lutfullina G. Lutfullin M. T. Akosah Y. A. Mardanova A. M. (2025). Genome sequence of *Bacillus subtilis* NGII: a rhizobacteria from potato plants. Microbiol. Resour. Announc. 15:e01025-25. doi: 10.1128/mra.01025-25, 41459961 PMC12896268

[ref25] Nishimura D. (2000). Repeatmasker. Biotech Softw. Inter. Rep. 1, 36–39. doi: 10.1089/152791600319259

[ref26] Ortiz A. Vaca J. Sansinenea E. Argentel-Martínez L. Peuelas-Rubio O. Kwon S. M. . (2026). *Bacillus subtilis* species complex: secondary metabolites, genomic insights, and metabolite-driven strategies for sustainable agriculture. J. Environ. Chem. Eng. 14:120631. doi: 10.1016/j.jece.2025.120631

[ref27] Petronaitis T. Simpfendorfer S. Hüberli D. (2021). “Importance of *Fusarium spp.* in wheat to food security: a global perspective.” In Plant Diseases and Food Security in the 21st Century, eds. Scott, P., Strange, R., Korsten, L., and Gullino, (M. L. Cham: Springer International Publishing), 127–159.

[ref28] Riseh R. S. Vatankhah M. Hassanisaadi M. Barka E. A. (2024). Unveiling the role of hydrolytic enzymes from soil biocontrol bacteria in sustainable phytopathogen management. Front. Biosci. (Landmark Ed) 29:105. doi: 10.31083/j.fbl2903105 38538262

[ref29] Schroeder J. W. Simmons L. A. (2013). Complete genome sequence of <>*Bacillus subtilis*</it> strain PY79. Genome Announc. 1:e01085-13. doi: 10.1128/genomea.01085-13, 24356846 PMC3868870

[ref30] Seemann T. (2014). Prokka: rapid prokaryotic genome annotation. Bioinformatics 30, 2068–2069. doi: 10.1093/bioinformatics/btu153, 24642063

[ref31] Sha Y. Wang Q. Li Y. (2016). Suppression of *Magnaporthe oryzae* and interaction between *Bacillus subtilis* and rice plants in the control of rice blast. Springerplus 5:1238. doi: 10.1186/s40064-016-2858-1, 27536521 PMC4971003

[ref32] Shao Z. Gu S. Zhang X. Xue J. Yan T. Guo S. . (2024). Siderophore interactions drive the ability of *Pseudomonas spp.* consortia to protect tomato against *Ralstonia solanacearum*. Hortic. Res. 11:uhae186. doi: 10.1093/hr/uhae186, 39247881 PMC11377186

[ref33] Srivastava S. Bombaywala S. Jakhesara S. Patil N. Joshi C. Purohit H. . (2022). Potential of camel rumen derived *Bacillus subtilis* and *Bacillus velezensis* strains for application in plant biomass hydrolysis. Mol. Gen. Genomics. 298, 361–374. doi: 10.1007/s00438-022-01987-y 36575347

[ref34] Wen Z. Yan N. Guo X. Liu Q. Chen J. (2026). *Paenibacillus polymyxa* 29-Y2: a promising endophytic biocontrol agent against wheat common bunt caused by *Tilletia foetida*. Plants 15:1072. doi: 10.3390/plants15071072, 41977731 PMC13075041

[ref35] Wick R. R. Judd L. M. Gorrie C. L. Holt K. E. (2016). Unicycler: resolving bacterial genome assemblies from short and long sequencing reads. PLoS Comput. Biol. 13:e1005595. doi: 10.1371/journal.pcbi.1005595, 28594827 PMC5481147

[ref36] Xu M. Wang Q. Wang G. Zhang X. Liu H. Jiang C. (2022). Combatting fusarium head blight: advances in molecular interactions between *Fusarium graminearum* and wheat. Phytopathol. Res. 4:37. doi: 10.1186/s42483-022-00142-0

[ref37] Xu Y. Wang Z. Wu J. Yue Y. Ren Y. Pan Y. . (2025). Keystone *Pseudomonas* species in the wheat phyllosphere microbiome mitigate *Fusarium* head blight by altering host pH. *Cell Host Microb*e. 33, 2052–2066.e4. doi: 10.1016/j.chom.2025.10.016, 41265441

[ref38] Yang Z. Zhang Q. Fu B. Zhang T. Feng Y. Zhao S. . (2025). A multi-enzyme producing *Bacillus subtilis* YCXW-01: isolation, genomic characterization, and potentials in tobacco stem degradation. Front. Microbiol. 16:1717865. doi: 10.3389/fmicb.2025.1717865, 41415817 PMC12709117

[ref39] Yu C. Liu X. Zhang X. Zhang M. Gu Y. Ali Q. . (2021). Mycosubtilin produced by *Bacillus subtilis* ATCC6633 inhibits growth and mycotoxin biosynthesis of *Fusarium graminearum* and *Fusarium verticillioides*. Toxins (Basel). 13:791. doi: 10.3390/toxins13110791, 34822575 PMC8620035

[ref40] Zeigler D. Prágai Z. Rodriguez S. Chevreux B. Muffler A. Albert T. . (2008). The origins of 168, W23, and other *Bacillus subtilis* legacy strains. J. Bacteriol. 190, 6983–6995. doi: 10.1128/JB.00722-08, 18723616 PMC2580678

[ref41] Zhang L. Shao L. Zhang B. Li Y. Wang Z. Song S. . (2026). Genome analysis and secondary metabolite characterization of *Bacillus velezensis* JZ as a potential biocontrol agent. Physiol. Mol. Plant Pathol. 141:103015. doi: 10.1016/j.pmpp.2025.103015

[ref42] Zhang C. X. Zhao X. Han F. Yang M. F. Chen H. Chida T. . (2009). Comparative proteome analysis of two antagonist *Bacillus subtilis* strains. J. Microbiol. Biotechnol. 19, 351–357. doi: 10.4014/jmb.0805.346, 19420989

[ref43] Zhu F. Wang J. Tian C. Xu Y. Gao Y. Zhang X. . (2026). Antifungal activity of volatile organic compounds produced by *Bacillus subtilis* GB519 against blast pathogen *Magnaporthe oryzae* in rice. Front. Microbiol. 17:1757473. doi: 10.3389/fmicb.2026.1757473, 41889645 PMC13013540

